# Associations between music training and language fluency on cognitive control and processing speed

**DOI:** 10.3389/fpsyg.2025.1662938

**Published:** 2026-01-09

**Authors:** Jennifer A. Bugos, Kya R. Laubisch, Angela Zhu, Jason Schmidt Avendaño, Judith B. Bryant

**Affiliations:** 1 School of Music, University of South Florida, Tampa, FL, United States; 2 Department of Psychology, University of South Florida, Tampa, FL, United States

**Keywords:** music training, bilingualism, multilinguals, executive functions, cognitive control

## Abstract

**Introduction:**

Scientific research has demonstrated that musical training and bilingualism contribute to enhanced executive functions (D’Souza et al., 2018). However, it is unknown whether fluency in three or more languages would confer additional cognitive benefits above bilingual and musician status. According to Patel’s OPERA Hypothesis, music shares many similar components of language and also contains a high level of task complexity and temporal elements (Patel, 2012). Previous studies suggest that music and language learning enhance executive functions in cognitive control mechanisms, improving the ability to inhibit irrelevant information, flexibly shift attention, and make quick decisions. This research sought to examine associations between bilingualism and language fluency in musicians as compared to multilingual non-musicians. Also, do adults who are fluent in three languages perform similarly to bilingual musicians? Our research assessed associations between multilingualism, cognitive performance, and musical training.

**Methods:**

Fifty-five participants (19 bilingual musicians, 18 bilingual non-musicians, 18 multilingual non-musicians) completed measures of visual motor processing and cognitive control in the visual and auditory domains.

**Results:**

Results of a two-way ANOVA revealed enhanced processing speed and cognitive control in visual and auditory domains for bilingual musicians as compared to bilingual and multilingual non-musicians.

**Discussion:**

Overall, multilingualism may not enhance executive functions beyond those acquired in music training.

## Introduction

Music training and language learning are complex tasks associated with generalized benefits in executive functions for children and adults (Román-Caballero et al., 2018; Tao et al., 2021). Formal music training is associated with learning to perform on a musical instrument, and bilingualism refers to learning how to speak and write fluently in a second language. There is evidence to suggest participation in complex activities such as language learning and music training individually benefit executive functions such as verbal fluency (Bak et al., 2014; Bugos, 2010; Patra et al., 2019), processing speed (Bugos et al., 2007; Grant and Dennis, 2016; Marton et al., 2016) and cognitive control (Gold et al., 2013; Jäncke et al., 2018; Bialystok, 2010). Based upon the effects of multilingualism and music training singularly on executive functions, we sought to examine whether bilingual musicians would demonstrate an additive effect on cognitive control and processing speed. While few researchers examine a potential additive effect of music training and language fluency (Moradzadeh et al., 2014; Schroeder et al., 2016), knowledge of potential benefits could increase the scope of cognitive interventions, especially for individuals with speech and language processing disorders (Amengual et al., 2013; Lahiri et al., 2020; Vik et al., 2018).

### Cognitive control and processing speed

Cognitive control, a primary component of executive functions in adults, refers to the regulation of one’s attention, behavior, thoughts, and emotions to resist internal impulses or external distractions, allowing for goal-directed responses (Miyake et al., 2000; Diamond, 2013). Music training has consistently been associated with improvements in cognitive control, with musicians demonstrating enhanced ability to suppress irrelevant information (Rogers and Metzler-Baddeley, 2024). However, research indicates that the relationship between language fluency and cognitive control in adult bilinguals is complex and varies across studies. While research by Chen et al. (2025) found enhanced performance by bilinguals on the Stroop task, the N-back task, and the switching task when compared to multilinguals, other studies found little evidence for a specific bilingual advantage, highlighting variability across studies (Dick et al., 2019; Duñabeitia et al., 2014).

Similar to results reported regarding cognitive control, research examining the role of bilingualism on information processing speed has frequently produced inconsistent findings (Chung-Fat-Yim et al., 2025). For instance, research by Grant and Dennis (2016) found bilingual participants completed tasks significantly faster across all conditions while maintaining accuracy, suggesting a potential advantage in processing speed. In contrast, research by Chung-Fat-Yim et al. (2025) found no significant difference between bilinguals and monolinguals using the Pattern Comparison test. To-date, few studies examine differences between bilinguals and multilinguals, and none include music training. Research in our lab has shown that adult musicians demonstrate enhanced information processing speed when compared to non-musicians (Bugos and Mostafa, 2011). Thus, we hypothesized that musical experience may confer an added benefit to the potential bilingual advantage.

### Music-language neural overlap

Music and language domains involve overlapping cognitive processes (e.g., perception, prediction, feedback, and correction). These processes engage neural areas that occasionally draw upon parallel neurocognitive resources (Patel, 2008). The OPERA hypothesis posits that under specific conditions – overlap, precision, emotion, repetition, and attention – music training can enhance auditory processing (Patel, 2011, 2014). When all these conditions align, neural changes are anticipated to lead to heightened sensory perception of acoustic features and improved cognitive performance in auditory selective attention. This enhancement is attributed to the shared cortical processing networks for language and music (Atherton et al., 2018; Schulze et al., 2012), evident in the auditory cortex (Rogalsky et al., 2011) and superior temporal gyrus (Merrill et al., 2012). Additionally, cross-domain overlap extends to syntactic processing, as proposed by the shared syntactic integration resource hypothesis (Patel and Daniele, 2003). Recent studies have unveiled this overlap in the processing of music and language, not only within the syntactic systems but also extending into the semantic domain (Schmidt Avendaño et al., 2023; Slevc et al., 2009; Hoch et al., 2011; Perruchet and Poulin-Charronnat, 2012; Steinbeis and Koelsch, 2008). These findings underscore the intricate relationship between music and language processing, highlighting shared neural substrates across different cognitive domains.

Overlapping qualities led us to compare the potential advantages from multilingualism and music training. Despite similarities in the benefits of music training and language learning, music training confers additional advantages not found in bilingualism. Studies of musicians demonstrated improvements in working memory, processing speed, and cognitive control (Criscuolo et al., 2019; Nutley et al., 2014). A longitudinal association study that measured cognitive performance over multiple time points during a 2-years span revealed positive effects of music training on visuo-spatial and verbal working memory, processing speed, reasoning ability, and associations with music practice and math performance (Nutley et al., 2014). In another study comparing musicians and non-musicians, Amer et al. (2013) found larger gray matter volume for musicians, particularly in late middle-aged to older adults, with professional musicians outperforming non-musicians on cognitive control tasks. Additionally, Criscuolo et al. (2019) found that professional adult musicians exhibited superior performance in working memory and attention tasks when compared to adult non-musicians.

Music training was shown to benefit auditory working memory among primary school children, particularly through a more extended instrumental training program (Roden et al., 2014). Furthermore, research suggests the potential for improved attention skills, verbal intelligence, and enhanced cognitive control performance in short-term music training interventions (Bugos and DeMarie, 2017; Huseynova et al., 2019; Moreno et al., 2011). For instance, a 14-weeks private piano tutoring program for 7–12-years-old children resulted in improved attention skills and enhanced reading time (Huseynova et al., 2019). Similarly, studies comparing preschool children receiving short-term music training to those receiving Lego training (Bugos and DeMarie, 2017) and visual art training (Moreno et al., 2011) demonstrated enhancements in cognitive control and verbal intelligence for children in the music training condition. Research suggests that music training increases many areas of executive functions that rely upon complex attention.

The positive links between higher cognitive performance in executive functions and music training extend across different age groups. Studies conducted with older adults who completed piano training, both in preliminary investigations (Bugos et al., 2007; Seinfeld et al., 2013) and randomized controlled trials (Bugos and Wang, 2022), highlight the cognitive benefits that music training can confer in adulthood. These studies revealed improvements in visual scanning, inhibitory control, and divided attention tasks within the piano instruction groups compared to control groups. In addition, one study examining sustainability found benefits to visual scanning and processing speed were maintained after training ceased, underscoring the enduring impact of music training on cognition (Bugos et al., 2007).

While music training supports executive functioning, emerging research explores the shared and distinct cognitive and neurophysiological mechanisms between music and language processing. Comparative analyses suggest that both domains recruit overlapping neural systems, particularly in semantic and syntactical processing (Lee et al., 2019; Yu et al., 2017). For example, target words accompanied by musical chords in a self-paced reading task contributed to larger syntactic violations in musicians compared to non-musicians as indexed by N400 amplitude (Schmidt Avendaño et al., 2023).

Further evidence for this shared processing comes from electrophysiological studies examining components like the Early Right Anterior Negativity (ERAN) and the N5. The ERAN, peaking around 250 ms, is elicited by violations of musical syntax and has been localized to inferior frontolateral cortical regions (Koelsch et al., 2000; Maess et al., 2001). The N5, emerging between 500 and 550 ms, is thought to reflect harmonic integration and exhibits a frontal scalp distribution similar to the linguistic N400 (Koelsch et al., 2005). Steinbeis and Koelsch (2008) reported that the ERAN was attenuated when music was presented concurrently with syntactic–but not semantic–language manipulations, reinforcing its role in syntax processing. However, they also found that the N5 was modulated by the semantic cloze probability of target words, suggesting that this component may reflect meaning-based integration across both domains. While some researchers argue that differences in the scalp distribution and timing of these components point to domain-specificity, the overall evidence supports at least partial overlap in the neural resources recruited by music and language.

Although music and language may utilize domain-specific resources, they also engage shared cognitive processes in complex meaning-making contexts. Supporting this interpretation, semantic priming paradigms have demonstrated that musical passages–such as excerpts from Mozart–can prime semantically related linguistic targets, much like traditional language-based priming (Koelsch, 2011; Koelsch et al., 2004).

### Bilingualism and musicianship

Bilingualism emerges as a potent factor in mitigating age-related cognitive decline, as evidenced by findings from a large-scale Lothian birth cohort study (Bak et al., 2014). Even after controlling for variables such as childhood intelligence, gender, and socioeconomic status, bilingualism had a protective effect, resulting in significant enhancements in reading, verbal fluency, and general intelligence when compared to monolinguals. Many studies underscore the cognitive benefits derived from language fluency (Bialystok and Poarch, 2014; Guzmán-Vélez and Tranel, 2015; Marian and Shook, 2012), thus drawing parallels to the advantages seen through music instruction, but less attention has been allocated to those fluent in multiple languages.

Functional imaging studies involving bilinguals, musicians, and monolingual non-musicians showed enhanced activation in anterior-posterior attention network while completing a non-spatial working memory task (e.g., N-Back) for all groups; however, heightened activity in known language-related areas was observed for bilinguals in the left supramarginal gyrus and left DLPFC (Alain et al., 2018). Findings suggest cognitive advantages for both bilinguals and musicians may be influenced by distinct neural networks reinforced through domain-specific training. For instance, Moradzadeh et al. (2014) examined task-switching, a crucial component of cognitive control and dual-task performance in monolingual musicians, bilingual musicians, bilingual non-musicians, and monolingual non-musicians. While musicians demonstrated heightened task-switching efficiency, no cognitive advantage was associated with language fluency. Bilingual and monolingual musicians exhibited similar performances across tasks. The literature presents a spectrum of findings: (1) musicians and bilinguals demonstrate comparable cognitive performance benefits in executive functions (Moreno et al., 2014; Schroeder et al., 2016), (2) research assessing young adults using a Simon measure reveals enhanced cognitive control in musicians, bilinguals, and bilingual musicians compared to participants lacking music or language fluency (Schroeder et al., 2016), and (3) studies investigating these associations report no bilingual advantage; however, a musician advantage is identified in working memory (D’Souza et al., 2018). Collectively, data suggests bilingualism is associated with enhanced executive functions; however, little is known about cognitive processing in multilinguals and the relationship between fluency and music training.

### Multilinguals

Studies of multilinguals suggest positive effects beyond those of bilinguals that may protect cognitive function (Hervais-Adelman et al., 2018; Perquin et al., 2013). Perquin et al. (2013) found associations between multilingualism and reduced cognitive impairment in older adults. Another Marian et al. (2013) examined cognitive control with a three language Stroop paradigm, found faster reaction times for congruent versus incongruent items for multilinguals. However, faster reaction times and increased accuracy were only found in the within-language condition, not between language conditions, suggesting processing costs when language presentation changed. However, other research in multilinguals offered contrasting results illustrating that linguistic distance between L2 and L3 can affect cognitive control related to either early-stage cognitive benefits obtained when learning a closely related L3 or reduce cognitive control performance due to higher levels of effort allocation necessary to learn a distant language (Nelyubina et al., 2025). However, Madrazo and Bernardo (2018) found enhanced performance for multilinguals on tasks that included response inhibition and interference suppression. Nevertheless, the role of multilingualism on cognition is not yet fully understood.

The primary objective of this research was to examine the associations between language fluency and music training. We aimed to determine whether there is an additive benefit to cognitive control and processing speed in a population of bilingual and multilingual adults. Research indicating differences between musicians and bilinguals does not merge these groups, nor consider music training as a language (D’Souza et al., 2018). This research is among the first to compare music training and language fluency in a three-group design: bilingual musicians, bilingual non-musicians, and non-musician multilinguals. Guided by the hypothesis that increased auditory experiences, or musical training might enhance executive functions in cognitive control, our investigation examines the relationship between linguistic proficiency, musical expertise, and cognitive processes.

## Materials and methods

### Participants

We recruited three groups of adults (19 bilingual musicians, 18 bilingual non-musicians, and 18 multilingual non-musicians) from a large urban university in the Southeastern United States. Screening criteria for all groups included right-handedness, those who reported no hearing or vision deficits, and those fluent in English as well as one additional language. Bilingual musicians (BMus) consisted of those who spoke two languages fluently, and who reported practicing a musical instrument for a minimum of 5 h per week and have played an instrument for a minimum of 5 years. Bilingual non-musicians (BNM) included those who spoke two languages fluently and reported having three or fewer years of formal musical experience and not currently engaged in music reading or performance. Criteria for multilinguals (MultiMus) included those who spoke three or more languages fluently and reported having less than 3 years of formal musical experience. Formal music experience was defined as private or group music lessons. Informed written consent was obtained in accordance with the policies of the University of South Florida Insitutional Review Board. Participants received financial compensation for their time in testing sessions.

The required sample size was calculated using G* Power 3.1 with a one-way ANOVA with a medium effect size (*f* = 0.25), an alpha level of 0.05, and a power of 0.80 (Kan, 2021). Fifty-five participants (BMus = 19, BNM = 18, MultiMus = 18) completed the study. Musicians recruited for this study had an average onset of music training of 9.22 years (SD = 2.66). Average age of onset for learning L2 for BMus (*M* = 6.63, SD = 4.12), BNM (*M* = 5.58, SD = 4.42), and MultiMus (*M* = 8.68, SD = 2.54).

Results of an ANOVA on demographic characteristics (see Table 1) showed no significant differences between groups in estimated IQ (WASI scores), gender, language proficiency, years of education, and language understanding (LEAP scores).

**TABLE 1:** Mean (±SD) demographic data.

Demographic factors	Group		
	BNM (*N* = 19)	BMus (*N* = 18)	MULTI (*N* = 19)
Age	20.95 (2.17)	22.11 (5.23)	21.68 (3.33)
Gender	9/10	9/9	8/11
Years of education	14.74 (1.59)	15.33 (2.77)	15.21 (1.48)
Age learned L2	5.58 (4.42)	6.63 (4.12)	8.68 (2.54)
Years of music training*	1.18 (1.18)	16.92 (8.30)	0.79 (1.02)
PRI	99.89 (11.28)	97.28 (11.52)	98.58 (13.87)
AMMA*	52.58 (6.54)	58.78 (7.21)	54.16 (8.02)
LEAP-Q	8.84 (0.69)	8.78 (1.26)	8.84 (0.96)

PRI, Perceptual Reasoning Index; AMMA, Advanced Measures of Music Audiation; LEAP-Q, Language Experience and Proficiency Questionnaire.

*Indicates statistical differences between the groups (*p* < 0.05).

### Procedure

All participants completed 1–2.5-h testing session in a quiet testing environment. Participants completed the Language Experience and Proficiency Questionnaire (LEAP-Q; Marian et al., 2007) to ensure language fluency. The LEAP-Q measure is commonly used in language studies and consists of nine demographic questions and seven language questions to evaluate language proficiency (Schroeder et al., 2016). For this study, we chose to use Item 3 in the language portion of the questionnaire to determine fluency status. Participant language fluency was also verified with a short performance task in which participants translated a paragraph into the second and/or third language (Interagency Language Roundtable, n.d.).

### Measures

The Wechsler Abbreviated Scale of Intelligence II (WASI- II; McCrimmon and Smith, 2012) was administered as an estimate of intelligence using two reliable non-verbal performance subtests, *Matrix Reasoning* and *Block Design* (*r* = 0.87–0.92). Raw scores were converted to age-adjusted scaled scores for the two-subtest version of the WASI. *T*-scores were derived for each independent measure and summed to form a composite. Composite scores were used to derive an estimate of intelligence standard score to characterize the sample (Wechsler, 1999). Since intelligence cannot be estimated accurately with verbal tasks in English for multilinguals due to differences in vocabulary, the two non-verbal subtests of the WASI were used to estimate intelligence (Schroeder et al., 2016). WAIS-IV Digit Coding and Symbol Search subtests were administered to compute (i.e., summation of scores) a Processing Speed Index (PSI) as these measures are commonly used in healthy and clinical populations (Wisdom et al., 2012; Erdodi and Abeare, 2019). The Digit Coding subtest includes a series of symbols to accompany numeric stimuli in which participants ascribe the appropriate symbols for numeric stimuli over 2 min. Performance on the Digit Coding subtest requires processing speed, working memory, visuospatial processing, and attention. The Symbol Search subtest includes two columns of symbols in which participants are asked to determine whether the symbol in one column matches any of those symbols presented in the other column. Participants are instructed to mark whether each symbol was replicated with a total of 2 s to complete the task. The Symbols Search subtest requires processing speed, visual scanning, and visuospatial processing (Wisdom et al., 2012).

To evaluate cognitive control in the verbal and musical domains, the Cued Color Word Stroop (CCWS; Perlstein et al., 2006) and the Musical Stroop Task (MST; Bugos et al., 2007) were administered. The computerized Cued Color Word Stroop consisted of 144 trials in four blocks of 36 pseudorandomized stimuli presented in the following block order: mixed, word, mixed, color. The measure was administered with E-Prime 3.0 on a MacBook Pro with Bootcamp. As in the standard Stroop task, participants are instructed to attend to either the ink color or the name of the word depending upon the instructions of the cue. Stimuli were presented in green, red, and blue. The cue was presented for 750 ms, followed by a 1,000-ms delay, and the stimulus was presented for up to 2500 ms or a response was made via a button box. Total errors and mean reaction times were collected over the four blocks. Items with no response were allotted the maximum response time. The Cued Color Word Stroop was reported to have test-retest reliabilities of 0.86, 0.82, and 0.73 for the individual task (Golden et al., 2000).

The computerized Musical Stroop Task (Bugos, 2010) included 240 trials with four blocks of pseudorandomized stimuli presented in the following order of blocks: Text, Mixed, Tune, and Mixed trials. Each trial included the first phrase of, “Mary Had a Little Lamb” or “Twinkle, Twinkle Little Star” presented with the last word or last two pitches congruent or modified. For instance, in the standard, “Twinkle, Twinkle Little Star,” the melody descends by one whole step at the end of the phrase. However, in the incongruent condition, the melody remained the same. Similarly, in the melody, “Mary Had a Little Lamb,” the last two notes at the end of the first phrase remain the same in the congruent condition. However, in the incongruent condition, notes descended by a whole step. Participants were instructed to respond to either the last word of the stimulus in the Text condition or the direction of the last two pitches (same or down) in the Tune condition. In the mixed blocks, participants were provided a visual cue of text or tune and asked to respond accordingly. Each trial consisted of a fixation of 1000 ms (a cross placed in the center of the screen), a 750-ms cue, a 1000-ms delay, and a 2000-ms sound file followed by a 2000-ms response duration. The Musical Stroop Task was chosen for its reliability in adults (*r* = 0.80; Bugos et al., 2007). Total errors and mean response times were calculated for each block (Text, Tune, or Mixed). Items with no response were allocated the maximum response time.

### Statistical analysis

A two-way mixed ANOVA was conducted for each Stroop measure (CCWS, MST) to examine (Block: Mix, Color Word; Mix, Text, Tune X Group: BNM, BM, MULTI) differences between reaction times and error rates. A separate one-way ANOVA with group as a fixed factor was conducted on PSI scores. For each analysis, Bonferroni *post hoc* comparisons were conducted to explore differences among three groups. Effect sizes for main effects and interaction effects are reported using partial η^2^.

## Results

We conducted two separate two-way ANOVAs as measures of cognitive control differed by modality (music and visual) (Table 2). Results of a two-way ANOVA on cognitive control in the auditory domain (MST) showed a significant main effect of Block for error rates, *F*(2, 51) = 215.16, *p* = 0.001, partial η^2^ = 0.81, and reaction times, *F*(2, 51) = 10.28, *p* = 0.001, partial η^2^ = 0.16. Our data showed a significant Group X Block interaction for error rates, *F*(4, 51) = 24.18, *p* = 0.001, partial η^2^ = 0.48, and for reaction times, *F*(4, 51) = 2.77, *p* = 0.031, partial η^2^ = 0.09. *Post hoc* comparisons using Bonferroni method showed significantly reduced errors and shorter reaction times for bilingual musicians compared to bilingual non-musicians and multilinguals.

**TABLE 2:** Mean (±SD) data.

Measures	Group		
	BNM (*N* = 19)	BMus (*N* = 18)	MULTI (*N* = 19)
CCWS errors	7.37 (4.810)	6.50 (4.878)	7.67 (6.765)
CCWS reaction time (ms)*	806.96 (103.499)	840.67 (159.150)	995.560 (196.796)
MST errors*	59.11 (20.870)	15.17 (13.021)	71.28 (34.776)
MST reaction time (ms)*	2013.807 (120.508)	1879.675 (78.878)	1871.894 (205.375)
TMTA (sec)*	25.40 (4.740)	31.10 (6.797)	31.76 (7.820)
TMTB (sec)*	58.10 (14.589)	58.61 (13.178)	62.22 (18.797)
WAIS-IV coding*	88.42 (11.744)	90.06 (11.507)	79.00 (13.412)
WAIS-IV symbol search	43.42 (7.066)	43.33 (6.183)	38.68 (6.807)

WAIS-IV, Weschler Adult intelligence Scale-IV; CCWS, Cue Colored Word Stroop; TMT, Trail Making Test; MST, Musical Stroop Task.

*Indicates statistical differences between the groups (*p* < 0.05).

**FIGURE 1:**
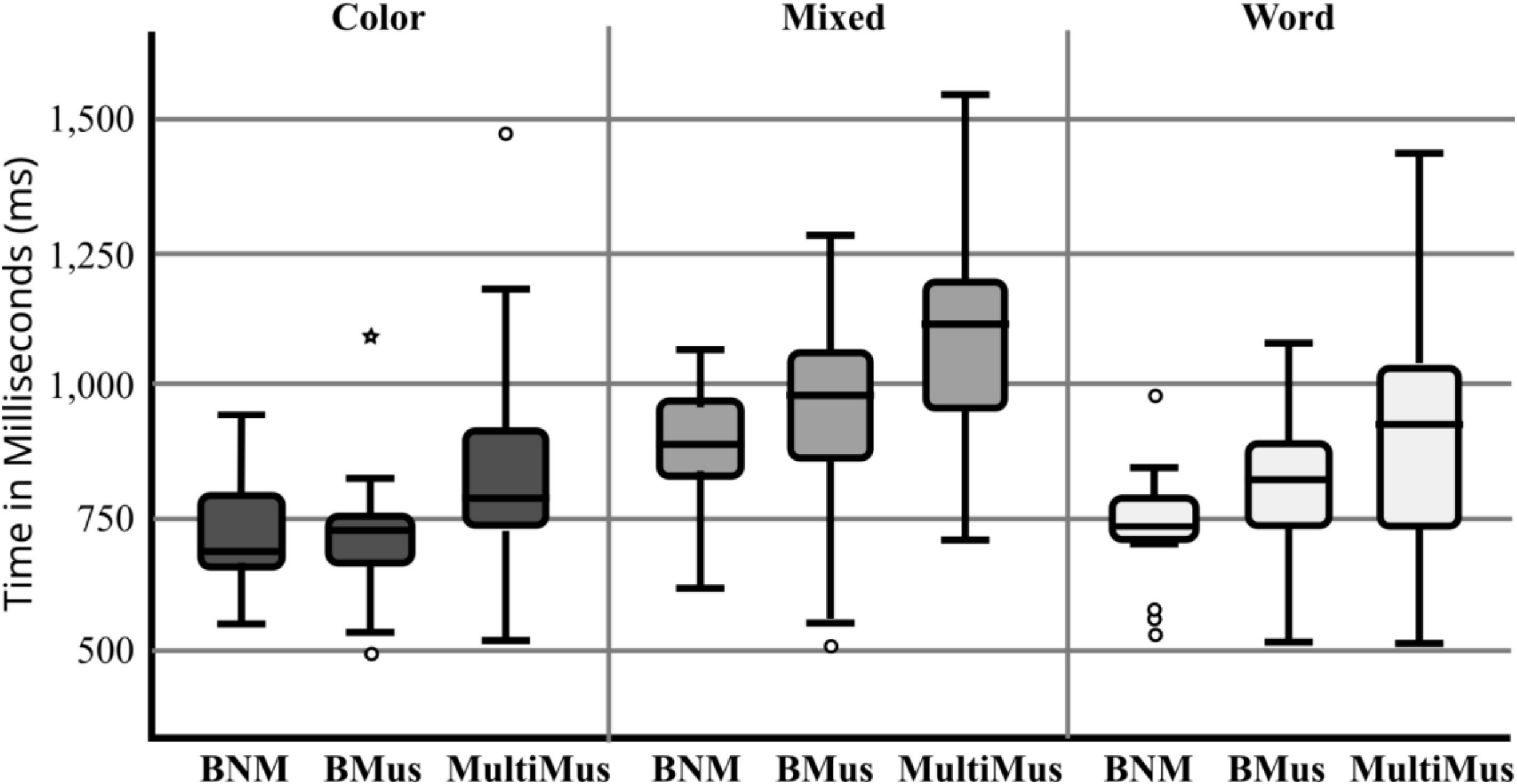
Reaction times (in ms.) by group for Cued Color Word Stroop.

We repeated this analyses for the CCWS (Figure 1) in the visual domain. Results of a two-way ANOVA on error rates and reaction times for the CCWS showed a significant main effect of Block for errors, *F*(2, 52) = 73.90, *p* = 0.001, partial η^2^ = 0.58, and for reaction times, *F*(2, 52) = 68.14, *p* = 0.001, partial η^2^ = 0.56. We did not see a significant Group X Block interaction for either error rates, *F*(4, 52) = 0.26, *p* = 0.90, nor reaction times, *F*(4, 52) = 1.64, *p* = 0.18. The pattern of results suggests similar performance regardless of language fluency or musical experience.

**FIGURE 2:**
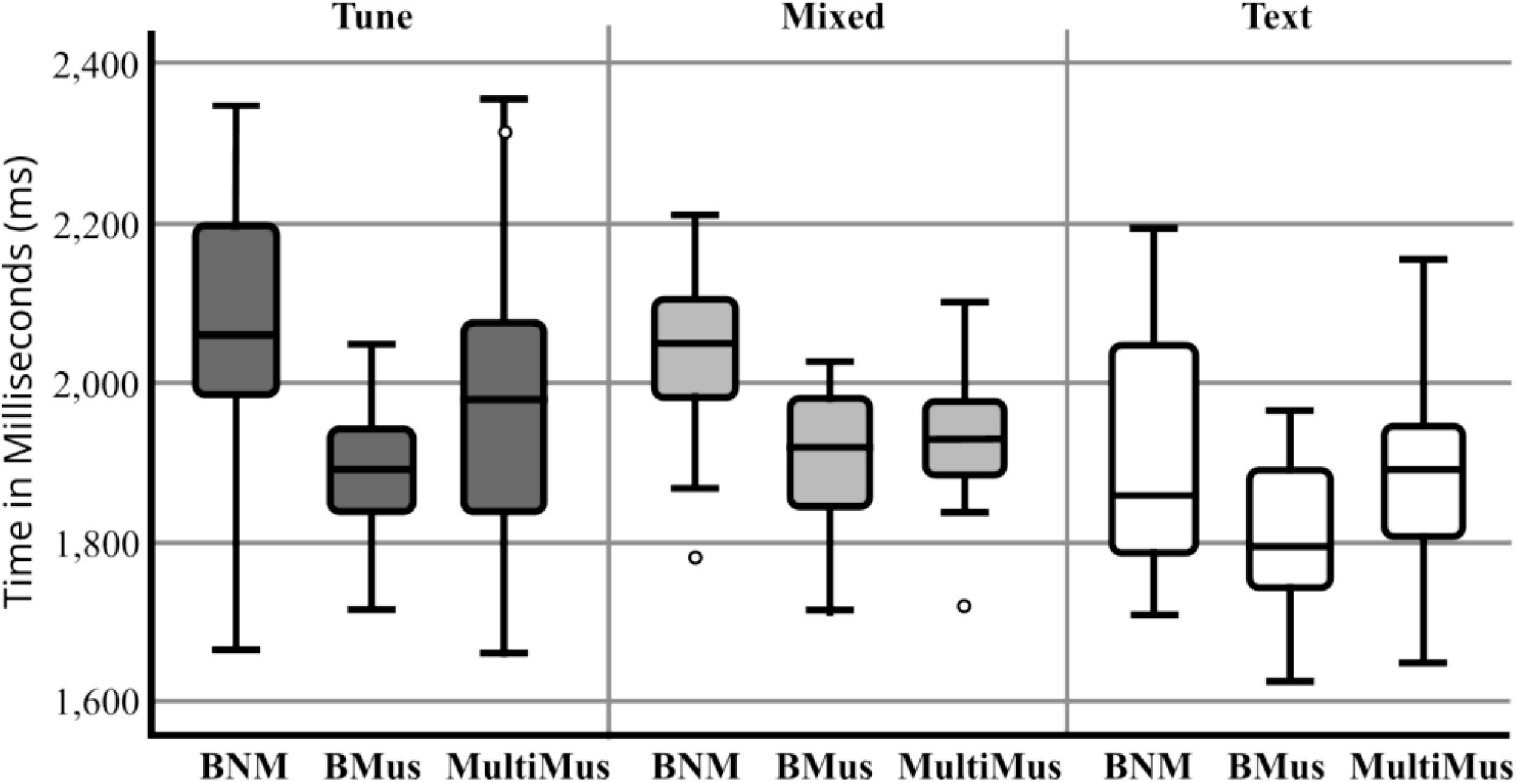
Reaction times (in ms.) by group for Music Stroop.

Data were contrasting when analyzing blocks on the MST (Figure 2). For the MST, we found differences in errors made on the Mixed blocks, *F*(2, 52) = 29.64, *p* = 0.0001, and Tune blocks, *F*(2,52) = 32.48, *p* = 0.0001. *Post-hoc* tests using the Bonferroni correction showed bilingual musicians performed less errors on Tune and Mixed blocks (*p* = 0.0001) compared to bilingual non-musicians and multilinguals. For MST reaction times, significant differences were found on the Tune, *F*(2,52) = 5.08, *p* = 0.010, partial η^2^ = 0.164, and Mixed Conditions, *F*(2,52) = 6.69, *p* = 0.003, partial η^2^ = 0.205 but not for the Text Condition *F*(2,52) = 1.69, *p* = 0.19 (Figure 2).

Results of an ANOVA on processing speed (Processing Speed Index) with group as a fixed factor revealed statistically significant differences (*p* < 0.05) in processing speed, *F*(2, 53) = 4.50, *p* = 0.016, partial η^2^ = 0.15. *Post hoc* comparisons using Bonferroni method revealed bilingual musicians had higher PSI indices compared to multilinguals or bilingual non-musicians.

Overall, results suggest slower processing speed and cognitive control by multilinguals compared to bilinguals. Similar performance was found between musicians and non-musicians in the visual domain; however, musicians demonstrated significantly enhanced performance in the auditory domain.

## Discussion

We investigated the associations between musicians and language fluency (bilinguals and multilinguals) on cognitive control and processing speed in young adults. Results suggest that long-term music training may be associated with benefits in these executive functions. Data are consistent with experimental research that demonstrates a causal relationship between music training and cognitive control (Seinfeld et al., 2013; Bayanova et al., 2024). Although we hypothesized there would be cognitive benefits for multilinguals, results show no additive effect of multilingualism in these areas of executive functions. This is consistent with previous research suggesting that multilinguals do not necessarily perform better on cognitive control tasks than bilinguals (Quinteros Baumgart and Billick, 2017). A possible explanation for these findings is that language processing might include additional levels of complexity that may be associated with different regions of the auditory cortices (Norman-Haignere et al., 2022).

Although there is controversy in the literature regarding code switching (Green and Wei, 2014), studies show that it allows bilinguals to separate languages more effectively, thus improving their executive functioning skills (Hofweber et al., 2020). However, our results showed that these benefits did not extend to multilinguals, specifically regarding processing speed. This may be attributed to the increased complexity that is brought about by being fluent in three or more languages, especially when taking into consideration additional tasks measuring executive functioning (Norman-Haignere et al., 2022). This is consistent with recent findings in multilinguals when compared to bilinguals on measures of cognitive control and processing speed such as the NIH Toolbox, Flanker and Inhibitory Control Measure (Chen et al., 2025).

Bilingual musicians made fewer errors than bilingual and multilingual non-musicians on the MST, which is consistent with previous studies that showed enhanced performance for musicians on auditory processing tasks (Musacchia et al., 2007). The reaction times for the MST suggest that auditory processing may not be as enhanced in bilingual non-musicians compared to bilingual musicians (Figure 2). In the CCWS, bilingual non-musicians and bilingual musicians demonstrated significantly faster reaction times compared to multilinguals. Our results showed that multilingualism does not necessarily produce an additive effect on processing speed and cognitive control. In contrast, we found no significant group differences in CCWS errors, which is to be expected for cognitive control (Mandikal Vasuki et al., 2017). Our results are consistent with neurophysiological data suggesting that there are shared resources amongst language and music domains within the brain (Li et al., 2023), thus explaining the enhanced auditory processing in bilingual musicians.

Results suggest that music training may enhance processing speed and auditory processing. Our findings are consistent with previous research that music training may enhance information processing speed in both visual and auditory domains (Bugos and Mostafa, 2011; Nan et al., 2016; Bugos et al., 2007) when compared to controls. Longitudinal research in music education and cognition showed that coordination developed during musical training relies heavily on rhythmic performance (Assaneo et al., 2024; Bugos, 2019). Assaneo et al. (2024) hypothesized that the refinement of rhythmic abilities in music education interventions may contribute to an overall boost in cognition.

Previous versions of a Musical Stroop Task required knowledge of note reading and solfege (Stewart, 2005; Grégoire et al., 2014). However, we chose to use the Musical Stroop Task for its accessibility for musicians and non-musicians. The reaction times for the MST suggest that auditory processing may not be as enhanced in bilingual non-musicians compared to bilingual musicians. Both bilingual groups (musicians and non-musicians) had reduced reaction times on CCWS when compared to the multilingual group. This may be due to a higher average age of onset for the third language in the multilingual non-musician group than the average age of onset for the second language in each group.

Patel’s (2012) OPERA hypothesis denoted that music and speech share acoustic features. According to the OPERA hypothesis, if overlap, precision, emotion, repetition, and attention are met, higher overall speech processing may result due to an increase in adaptive plasticity in neural networks (Patel, 2012). This aligns with our findings highlighting that language processing may be enhanced by music training. However, even though multilingualism has benefits as shown in other studies (Dolas et al., n.d.; Fürst and Grin, 2023; Kroll and Dussias, 2017), the present results show that multilingualism does not enhance executive functions beyond that of bilinguals.

### Limitations

We found associations between language fluency and music training; however, there are a few limitations. While this study was not causal in nature, our results make a key contribution to understanding how music and language learning transfer to executive functions. Results highlight the role of music training and bilingualism on cognitive control, necessary for generalized cognitive performance and selective attention (Vibell et al., 2021). Additionally, improved task switching and dual-task performance has been associated with musical-training (Moradzadeh et al., 2014). Our results support the OPERA Hypothesis, given that musical training enhances multitasking abilities (Patel, 2012).

Our study is unique as we utilized a rigorous screening process, ensuring that all participants were fluent in the spoken languages (LEAP-Q). Based upon our enrollment criteria, we could not examine a group of multilingual musicians. Additional studies are needed to further explore the benefits associated with multilingualism in a true experimental design with multilingual musicians to better understand potential additive benefits of music training and language learning with additional groups of monolingual speakers.

Since tonal language speakers have higher music aptitude (Bidelman et al., 2013), one limitation of this study included that five participants in this study spoke tonal languages and thus, it is unclear if this had any bearing on these results. Research suggests that even amongst Mandarin speakers, musicians demonstrated an enlarged electrical response to lexical tone changes and exhibited significantly faster discrimination performance than non-musicians, resulting in better lexical tone perception even in a tonal language (Nan et al., 2016).

Since most of the musicians within the study began formal music instruction around age nine on various instruments, further research is necessary to isolate the role of early music training during the sensitive period (i.e., prior to age seven) as other studies associated cognitive benefits (Moreno et al., 2015) and structural differences in the hippocampus, amygdala, and basal ganglia with early music training (Vaquero et al., 2016; Bailey and Penhune, 2013). Future studies should consider the age of onset and differentiate the type of instrumental training.

## Conclusion

The present work examined associations between multilingualism, cognitive performance, and music training. It was found that bilingual musicians performed better on executive functions and processing speed in auditory and visual domains as compared to multilingual and bilingual non-musicians. Collectively, associational studies along with longitudinal studies suggest that musical training may prepare the mind for learning through enhanced executive functions (Miendlarzewska and Trost, 2014). Furthermore, results of the study indicated that although music training may help to enhance processing speed, multilingualism may not necessarily produce an additive effect on cognitive control and processing speed, consistent with previous results (Bugos and Mostafa, 2011; Quinteros Baumgart and Billick, 2017). Although more research is necessary to explore the mechanisms behind these findings, our results ultimately clarify that fluency in three or more languages may not confer additional cognitive benefits above bilingual and musician status.

## Data Availability

The datasets presented in this study can be found in online repositories. The names of the repository/repositories and accession number(s) can be found below: https://osf.io/g8nkq/.
